# The Change of Intra-cerebral CST Location during Childhood and Adolescence; Diffusion Tensor Tractography Study

**DOI:** 10.3389/fnhum.2016.00638

**Published:** 2016-12-20

**Authors:** Yong M. Kwon, Hyeok G. Kwon, Jessica Rose, Su M. Son

**Affiliations:** ^1^Department of Physical Medicine and Rehabilitation, College of Medicine, Yeungnam UniversityTaegu, South Korea; ^2^Department of Orthopaedic Surgery, School of Medicine, Stanford UniversityStanford, CA, USA

**Keywords:** diffusion tensor, corticospinal tract, aging, maturation, children, cortex

## Abstract

**Objectives:** Corticospinal tract (CST) is the most important tract in motor control. However, there was no study about the change of CST location with aging. In this study, using diffusion tensor tractography (DTT), we attempted to investigate the change of CST location at cortex, corona radiata (CR) and posterior limb of internal capsule (IC) level with aging in typically developing children.

**Methods:** We recruited 76 healthy pediatric subjects (range; 0–19 years). According to the result of DTT, the location of CST at cortex level was classified as follows; prefrontal cortex (PFC), PFC with Premotor cortex (PMC), PMC, PMC with primary motor cortex (M1), M1, M1 with Primary sensory cortex (S1). Anterior-posterior location (%) of CSTs at CR and IC level was also assessed.

**Results:** DTT results about CSTs of 152 hemispheres from 76 subjects were obtained. The most common location of CST projection was M1 area (58.6%) including PMC with M1 (25.7%), M1 (17.8%), and M1 with S1 (15.1%). The mean age of the projection of CST showed considerably younger at anterior cortex than posterior; (PFC; 4.12 years, PFC with PMC; 6.41 years, PMC; 6.72 years, PMC with M1; 9.75 years, M1; 9.85 years, M1 with S1; 12.99 years, S1; 13.75 years). Spearman correlation showed positive correlation between age and the location of CST from anterior to posterior brain cortex (*r* = 0.368).

**Conclusion:** We demonstrated that the location of CST projection is different with aging. The result of this study can provide the scientific insight to the maturation study in human brain.

## Introduction

The corticospinal tract (CST) is the major neural tract in motor function. The CST is known to be involved mainly in functional use of distal extremities such as fine motor coordination (Martin, 2005; Baek et al., 2013; Yeo et al., 2014; Chang et al., 2015; Kim et al., 2015). Main cortical origin of CST is the primary motor cortex (M1), but previous studies reported various anatomical origins of CST in cortex level, such as parietal cortex, supplementary motor area (SMA) or premotor cortex (PMC; Russell and Demyer, 1961; Dum and Strick, 1991; Lemon and Griffiths, 2005; Martin, 2005; Seo and Jang, 2013). These various origins of CST appear to be related to various motor functions. They also can be the scientific basis upon perilesional reorganization after M1 injury which is one of motor recovery mechanisms of the CST.

Several previous studies reported that CST with different cerebral origin has different characteristics of the motor function, but most studies were postmortem studies which investigated animal brain instead of human brain (Russell and Demyer, 1961; Galea and Darian-Smith, 1994; Oudega et al., 1994; Maier et al., 1997; Dum and Strick, 2002; Martin et al., 2004; Lemon and Griffiths, 2005; Seo and Jang, 2013). Recent development of diffusion tensor tractography (DTT) allows three-dimensional reconstruction of neural tract in brain *in vivo*. DTT could define the anatomical location and somatotopic arrangement of CST in several levels of brain, including subcortical level such as internal capsule (IC) or corona radiata (CR) as well as cortical level (Kumar et al., 2009; Han et al., 2010; Hong et al., 2010; Kwon et al., 2011; Seo et al., 2012).

However, there is no study about the change of intra-cerebral CST location during childhood and adolescence. In the current study, we investigated anatomical change of the CST with aging in typically developing children and adolescents using DTT.

## Materials and Methods

### Subjects

Seventy-six typically developing right handed children and adolescent subjects (45 males, 31 females; mean age, 8.77 ± 5.08 years; range, 0–19 years) with no history of brain trauma or neurologic, psychological disease including delayed development were recruited. All subjects underwent evaluation by a neurologist and were diagnosed as normal healthy subjects. Parents of all subjects included in this study volunteered for this study. Written informed consent was obtained from the parents of all subjects. The study was approved by the institutional review board at our hospital.

### Diffusion Tensor Image

DTI data were obtained by using a Synergy-L sensitivity encoding six-channel head coil on a 1.5T GyroscanIntera system (Philips, Best, The Netherlands) equipped with single-shot echo-planar imaging. For each of the 32 non-collinear diffusion-sensitizing gradients, we acquired 67 contiguous sections parallel to the anterior/posterior commissure line. Imaging parameters were as follows: acquisition matrix = 96 × 96, reconstructed to matrix = 128 × 128, FOV = 221 mm × 221 mm, TR = 10,726 ms, TE = 76 ms, parallel imaging reduction factor (SENSE factor) = 2, EPI factor = 49, *b* = 1000 s/mm^2^, NEX = 1, and a section thickness of 2.3 mm (acquired voxel size, 1.73 mm × 1.73 mm × 2.3 mm). The Oxford Center for Functional Magnetic Resonance Imaging of the Brain (FMRIB) Software Library^1^ was used for correction of head motion effect and image distortion due to eddy currents. For analysis of the CST, the first ROI was placed on the CST portion of the ponto-medullary junction (blue color) and the second ROI on the CST portion of the anterior portion of the midpons (blue color). We reconstructed CST using DTI-Studio software with a 0.2 of fractional anisotropy and tract turning-angle of <60° (CMRM, Johns Hopkins Medical Institute, Baltimore, MD, USA).

### Measurement of the Location of the CST at the Level of the Cortex

**FIGURE 1:**
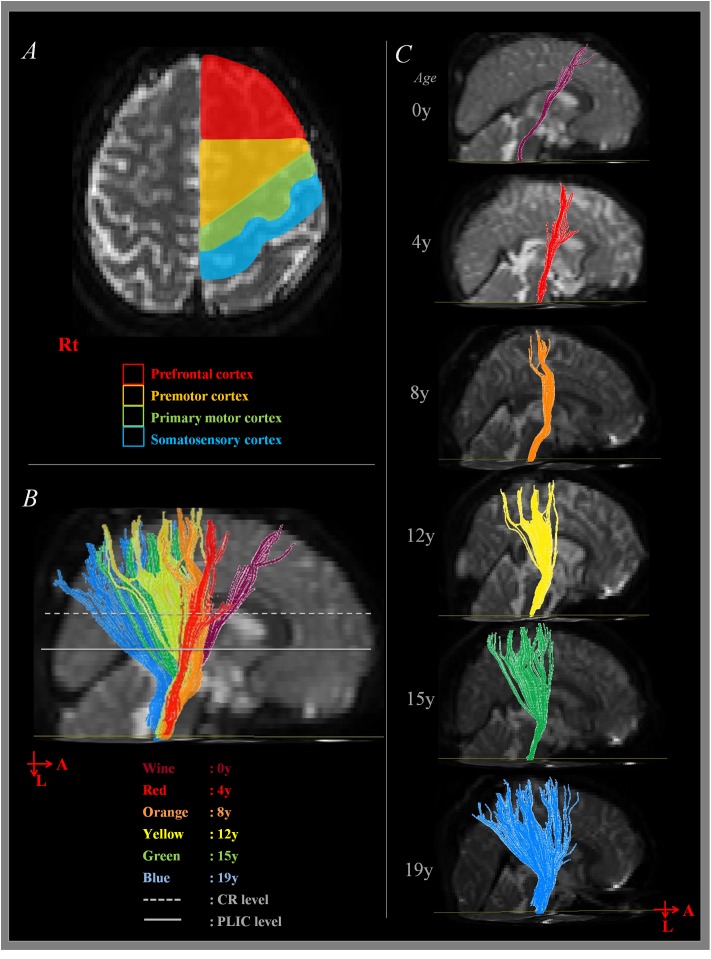
**Measurement of corticospinal tract (CST) at cortex level. (A)** Shows the boundary of cortex area for CST analysis according to the direction of anterior-posterior. **(B)** Shows the sagittal image of summated results of CST analysis. **(C)** Shows sagittal image of CST analysis according to the age. A, anterior; L, left; Rt, right; CR, corona radiata; PLIC, posterior limb of internal capsule.

Brain cortex areas were divided into four area as follows: (1) Prefrontal cortex [PFC] (BA 8 - the anterior boundary: the second bank of dorsolateral prefrontal cortex from pre-central sulcus, the posterior boundary: the anterior margin of BA 6, the medial boundary: the midline between the right and left hemispheres, and the lateral boundary: the inferior frontal sulcus) (Greenstein and Greenstein, 2000; Mayka et al., 2006; Yoshor and Mizrahi, 2012). (2) PMC (BA 6 - the anterior boundary: the line drawn through the anterior commissure perpendicular to the anterior commissure–posterior commissure line, the posterior boundary: the pre-central sulcus, the medial boundary: the midline between the right and left hemispheres, and the lateral boundary: the lateral sulcus; Greenstein and Greenstein, 2000; Mayka et al., 2006; Yoshor and Mizrahi, 2012). (3) Primary motor cortex [M1] (BA 4 – the anterior boundary: the pre-central sulcus, the posterior boundary: the central sulcus, the medial boundary: the midline between the right and left hemispheres, and the lateral boundary: the lateral sulcus; Greenstein and Greenstein, 2000; Mayka et al., 2006; Yoshor and Mizrahi, 2012). (4) Primary sensory cortex [S1] (BA 1, 2, and 3 – the anterior boundary: central sulcus, the posterior boundary: post-central sulcus, the medial boundary: the midline between the right and left hemispheres, and the lateral boundary: the lateral sulcus; Greenstein and Greenstein, 2000; Yoshor and Mizrahi, 2012) (**Figure 1A**). According to the projection site of analyzed CST at level of cortex, CSTs were classified into PFC, PMC, M1, and S1 cortex group.

### Measurement of the Location of the CST at the Level of the CR

**FIGURE 2:**
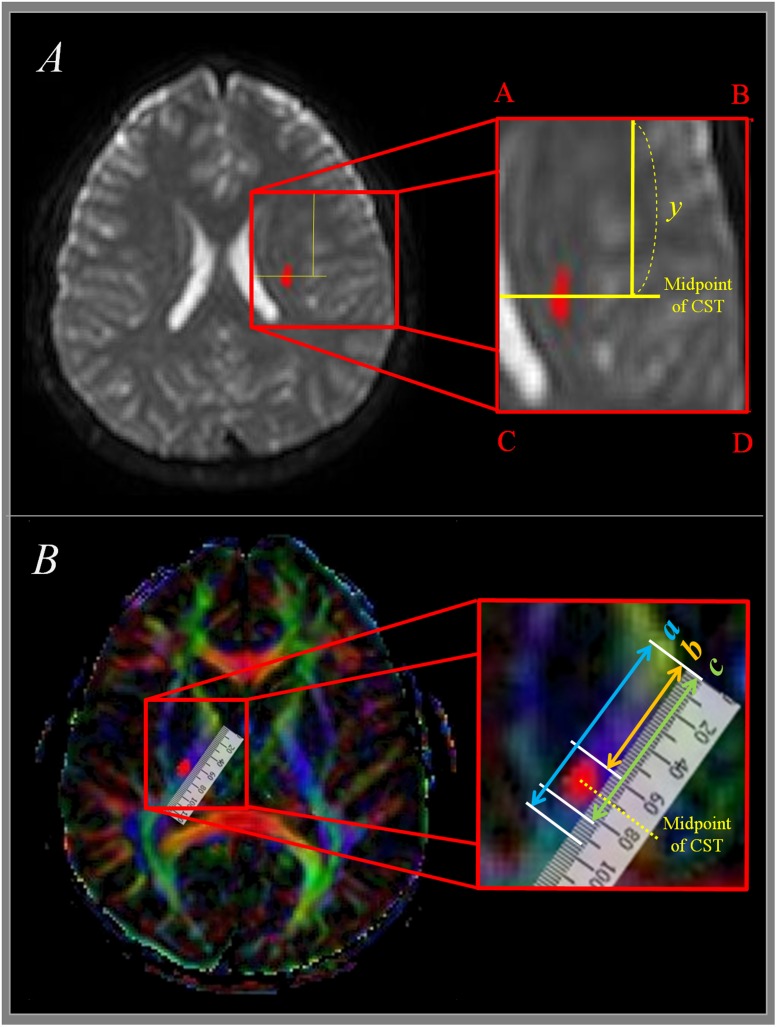
**Measurement of CST at corona radiata and internal capsule level. (A)** Shows measurement of CST at corona radiata level. *Y* means anterior-posterior location from anterior boundary (AB) to midpoint of CST. **(B)** Shows measurement of CST at posterior limb of internal capsule level. The total length of the posterior internal capsule is expressed as ‘a,’ the distance between the anterior margin of posterior internal capsule and the anterior margin of CST is expressed as ‘b,’ and the distance between the anterior margin and the midpoint of CST is expressed as ‘c.’ CST, corticospinal tract.

We measured the antero-posterior (AP) locations of pixels in the CST in CR where the first axial image seen in the septum pellucidum and body of fornix from the vertex. As shown in **Figure 2A**, the line AC passing through the most medial point of the lateral ventricle wall was defined as the medial boundary, and the line BD passing the most lateral point of the cerebral cortex was defined as the lateral boundary. AB, which passes through the most anterior point of the lateral ventricle, was defined as the anterior boundary, and CD, which passes through the most posterior point of the lateral ventricle was defined as the posterior boundary. AP location (Y) of each pixel in the CST was defined as below (Han et al., 2010).

$$Y=\frac{\mathrm{The distance from AB to the pixel}}{\mathrm{The lenght of AC}}\times 100$$

### Measurement of the Location of the CST at the Level of the IC

The pathway of the CST was determined same as the manner of Kumar et al. (2009); the IC of posterior limb, thalamus, putamen, and globus pallidus were showed at the horizontal plane (1) the anterior margin of IC was defined as the medial apex of the globus pallidus, and the posterior margin of IC was defined as the posterior apex of the putamen. The total length of the IC (*a* in **Figure 2B**), the distance between the anterior margin of IC and the anterior margin of CST (*b* in **Figure 2B**), and the distance between the anterior margin of the IC and the posterior margin of the CST (*c* in **Figure 2B**) were measured. From these measurements, the distance from the anterior margin of PLIC up to the midpoint of the CST was determined. A percentage value for the midpoint of the CST within the IC, where 0% is the anterior margin and 100% is the posterior margin in a given axial plane was measured.

### Statistical Analysis

The Statistical Package for the Social Sciences software (Version 15.0; SPSS, Chicago, IL, USA) was used for data analysis. For statistical comparison of the mean age for each CST location in cortex level, one-way analysis of variance (ANOVA) with *post hoc* Levene test was used. Spearman’s correlation coefficient was used for statistical significance of correlation between age and CST location in brain cortex. For statistical analysis of correlation between age and CST location in CR and IC level, Pearson’s correlation coefficient was used. The level of significance was set to *p* < 0.05. Each tract in all subjects was reconstructed twice by one evaluator (Kwon YM) with 1 week interval between examination. The location of analyzed tract in the cortex, CR and IC level was checked and accuracy of the test–retest reliability was 0.94, showed a high rate of reliability.

## Result

One hundred fifty-two CSTs from 76 subjects were obtained (**Table 1**).

**Table 1:** Age distribution of subjects.

Age group (*n*)	Age (years)	*n* (male:female)	Age group (*n*)	Age (years)	*n* (male:female)
0–4 years (17)	0	4 (3:1)	10–14 years (25)	10	5 (3:2)
	1	4 (2:2)		11	5 (3:2)
	2	3 (2:1)		12	5 (3:2)
	3	4 (2:2)		13	5 (3:2)
	4	2 (2:0)		14	4 (2:2)
5–9 years (25)	5	5 (3:2)	15–19 years (10)	15	3 (1:2)
	6	6 (4:2)		16	2 (1:1)
	7	4 (1:3)		17	1 (1:0)
	8	4 (2:2)		18	2 (2:0)
	9	6 (4:2)		19	2 (1:1)

**FIGURE 3:**
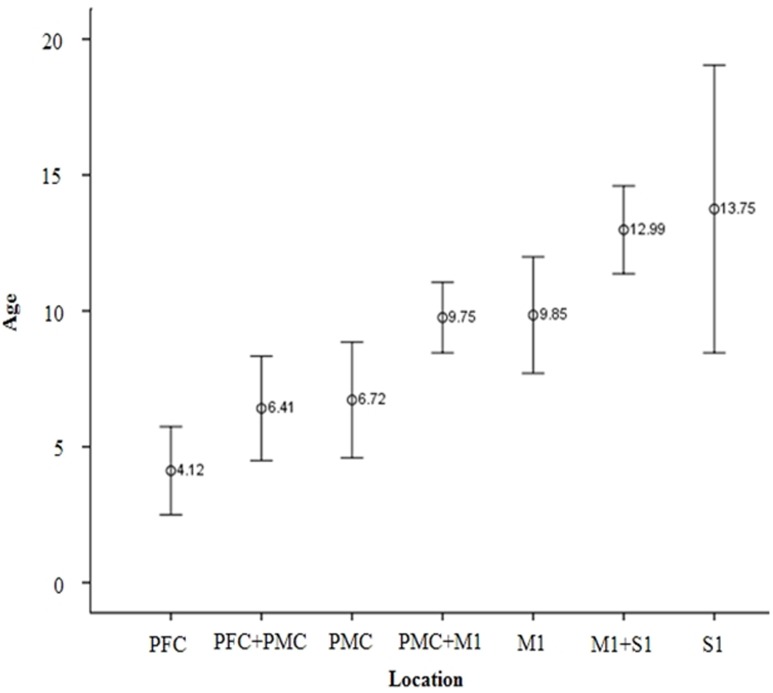
**Mean age of CST projection according to brain cortex area**. With aging, the main location of CST is changed from anterior to posterior direction in sagittal section. Among mean age of each age group, there were significantly different results (*p* < 0.05). PFC, Prefrontal cortex; PMC, Premotor cortex; M1, Primary motor cortex; S1, Primary sensory cortex

**FIGURE 4:**
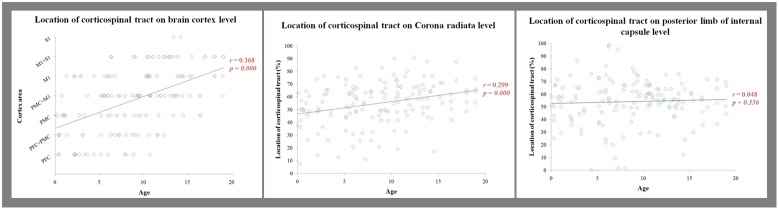
**Results of correlations**.

The overall proportion of main projection site in brain cortex was as follows: PFC (11.2%), PFC with PMC (12.5%), PMC (16.4%), PMC with M1 (25.7%), M1 (17.8%), M1 with S1 (15.1%), and S1 (1.3%). Generally, M1 area was the most common site (58.6%) among the four areas in cortex level (PFC, 23.7%; PMC, 54.6%; S1, 16.4%). Interestingly, main location in cortex level showed anterior to posterior direction in sagittal section with aging (**Figures 1B,C**). That is, the mean age of PFC showed the youngest age with 4.12 ± 3.16 years, the mean age of PFC with PMC was 6.41 ± 3.99 years, PMC was 6.72 ± 5.16 years, PMC with M1 was 9.75 ± 4.01 years, M1 was 9.85 ± 5.43 years, M1 with S1 was 12.99 ± 3.73 years and S1 was 13.75 ± 0.59 years (**Figure 3**). There were significant differences among the mean age of each group (*p* < 0.05; **Table 2**). The mean age of PFC showed significant differences with that of PMC + M1 (*p* = 0.000), M1 (*p* = 0.001), M1 + S1 (*p* = 0.000), and S1 (*p* = 0.000) group (*p* < 0.05). The mean age of PFC + PMC showed significant differences with M1 + S1 (*p* = 0.000) and S1 (*p* = 0.000) group (*p* < 0.05). The PMC showed significant differences with M1 + S1 (*p* = 0.000), and S1 (*p* = 0.000) group (*p* < 0.05). The PMC + M1 showed significant differences with PFC (*p* = 0.000), M1 + S1 (*p* = 0.049), and S1 group (*p* = 0.008; *p* < 0.05). The M1 showed significant differences with PFC (*p* = 0.001) and S1 (*p* = 0.047) group (*p* < 0.05). The mean age gap between PFC, PMC, M1 and S1 showed the longest age gap between M1 and S1 (mean age gap: 3.9 years) and followed by PMC and M1 (mean age gap: 3.13 years) and the shortest between PFC and PMC (2.6 years). Results of correlation analysis between age and projection area of brain cortex showed weakly positive correlation from anterior to posterior cortex [Spearman *r* = 0.368, *p* = 0.000] (**Figure 4**).

**Table 2:** Difference of mean age between corticospinal tract projection sites in the cortex level.

	Average	*SD*		Average	*SD*	*p*-value
PFC	4.12	3.16	PFC + PMC	6.41	3.99	0.746
			PMC	6.72	5.16	0.655
			PMC + M1	9.75	4.01	0.000^∗^
			M1	9.85	5.43	0.001^∗^
			M1 + S1	12.99	3.73	0.000^∗^
			S1	13.75	0.59	0.000^∗^
PFC + PMC	6.41	3.99	PMC	6.72	5.16	1.000
			PMC + M1	9.75	4.01	0.101
			M1	9.85	5.43	0.308
			M1 + S1	12.99	3.73	0.000^∗^
			S1	13.75	0.59	0.000^∗^
PMC	6.72	5.16	PMC + M1	9.75	4.01	0.297
			M1	9.85	5.43	0.561
			M1 + S1	12.99	3.73	0.000^∗^
			S1	13.75	0.59	0.000^∗^
PMC + M1	9.75	4.01	M1	9.85	5.43	1.000
			M1 + S1	12.99	3.73	0.049^∗^
			S1	13.75	0.59	0.008^∗^
M1	9.85	5.43	M1 + S1	12.99	3.73	0.346
			S1	13.75	0.59	0.047^∗^
M1 + S1	12.99	3.73	S1	13.75	0.59	1.000

PFC, Prefrontal cortex; PMC, Premotor cortex; M1, Primary motor cortex; S1, Primary sensory cortex; SD, standard deviation. *Significant difference among the mean age of each group, *p* < 0.05.

For the CR level, the CST showed weakly positive correlation between aging and main location which showed anterior to posterior direction [Pearson *r* = 0.299, *p* = 0.000] (**Table 3**, **Figure 4**). However, for the IC level, there was no significant correlation between aging and location [Pearson *r* = 0.048, *p* = 0.556] (**Table 3**, **Figure 4**).

**Table 3:** Location of corticospinal tract at corona radiata and internal capsule level according to cortex projection area.

	Prefrontal	PFC + PMC	PMC	PMC + M1	M1	M1 + S1	S1	Total average	*SD*
Corona radiata (%^∗^)	33.46	45.50	48.55	58.50	64.15	69.38	71.2	55.08	16.47
IC (%^∗^)	34.10	50.63	46.13	58.02	64.94	61.98	65.7	54.13	17.25

PFC, Prefrontal cortex; PMC, Premotor cortex; M1, Primary motor cortex; S1, Primary sensory cortex; IC, internal capsule; SD, standard deviation. *Average locations of corticospinal tract (%, 0~100).

## Discussion

In the current study, we investigated the anatomical change of CST location with aging in normal healthy childhood and adolescent subjects using DTT. Age showed significant correlation with the CST location at cortex and CR level from anterior to posterior direction, but no significant correlation with that in IC level.

To the best of the knowledge, there were only two studies about the change of anatomical location of CST. Oudega et al. (1994) reported in rat model that early in development, corticospinal neurons are distributed throughout much of the frontal and parietal lobes, but their distribution is later restricted to the posterior frontal and parietal lobes. In 2009, by diffusion tensor imaging (DTI) study by Kumar et al. (2009) reported about origin and course of the CST in 42 healthy children. In their study, there was significant difference between children with CST originating from both pre- and post-central gyri and the children with CST origin from pre-central gyri only, that is, the mean age of pre-central gyri origin only group was significantly younger (5.8 ± 4.3 years) than that of pre-and post-central gyri group (11.1 ± 4.1 years). These results correspond with the results of ours in that CST location changes from anterior to posterior direction with aging.

It is well-known that there is a somatotopical anterior-to-posterior (AP) arrangement of the CST at the CR (Tohgi et al., 1996; Kim and Pope, 2005; Han et al., 2010). In the current study, we found that the CST descended at approximately 55.1% of the AP direction at the CR level (**Table 2**). In the previous studies, there was similar results about the CST location at CR level; posterior half of the CR where the first axial image seen in the septum pellucidum and body of fornix (Newton et al., 2006). Upon IC level, previous DTI studies reported the CST from the M1 located at the posterior portion of the IC in adult brain (Tohgi et al., 1996): 31–68 years; (Kim and Pope, 2005): 23–57 years; (Newton et al., 2006; Zarei et al., 2007). In another study, Kim et al. (2008) demonstrated that the CST progressively shifted position into the posterior half of the posterior limb of the IC in the caudal portion. Our results in adolescents (older than 13 years) also coincide with this study that who are older than 13 years usually include posterior brain cortex such as M1 or S1 area and posterior half of CR. However, the somatotopic organization of the CST at the IC has been a matter of considerable debate. Traditional concept was that, the CST pass through the anterior one third of the IC (Charcot and Hadden, 1883; Holodny et al., 2005). Nowadays many subsequent studies have challenged this view and have suggested that the CST may be localized in the posterior portion of the IC (Kim et al., 2008; Kumar et al., 2009). However, in the current study, CST at IC showed only trend AP location according to aging with no significance. The reason might be shorten length and compact area of posterior limb of IC level than CR area. Direct comparison, however, would be impossible mainly due to different age groups. Another reason would be possibility of different measurement because earlier studies did not define the exact boundary of the posterior limb of the IC and their analyzed area, or did not declare the axial cutting level.

For the cortical origins of CST, there were several previous studies in animals or human subjects. Maier et al reported that CST projection from mainly M1 with SMA in the macaque monkey (Maier et al., 1997). Russell et al reported that CST originated from various cortex areas including M1, PMC and parietal lobe in the macaque monkey (Russell and Demyer, 1961). Martin et al reported that CST originated from not only M1 but also PMC in cats’ brain (Martin et al., 2004). Other studies also reported that CST is originated from mainly M1 but also from several other areas (Galea and Darian-Smith, 1994; Oudega et al., 1994; Dum and Strick, 2002; Lemon and Griffiths, 2005; Seo and Jang, 2013)

It is known that each area in adult brain plays a characteristic role: M1, executive function of movements; SMA, planning and coordination of internally guided movements sequences; PMC, sensory guidance of movement; S1, descending control of afferent sensory input from movements (Jane et al., 1967; Galea and Darian-Smith, 1994; Lemon et al., 2002; Lemon and Griffiths, 2005). Seo and Jang (2013) reported interesting result about different characteristics of various CSTs with different cortical origin from PMC, SMA, M1, and S1 using DTI. They compared the diffusion parameters of CSTs with different cortical origins in healthy subjects, showing significantly different FA values of CSTs from the PMC and SMA compared to those from M1 and S1. However, no significant differences of FA values were observed between CSTs from the M1 and S1. In spite of the different study design from ours, there is similar point between their study and ours. Their result showed similar characteristics between M1 and S1 compared to PMC and SMA. In our study, all CSTs projected from S1 area also projected from M1 area except only two hemispheres which might due to their similar characteristic.

Exact developmental process for each cortical area in human brain is not understood. However, it is well known that M1 is main area for CSTs in adults. The traditional explanation of the motor development is that inhibition of subcortical or reflexive movement is the main function of cortical centers for voluntary motor function in early stages of life. After then, with aging, voluntary motor function is facilitated under higher level of control (Thelen, 1995). During early period, children cannot use fingers properly compared to older children. Young children can reach or grasp the objects, but they show clumsiness during manipulation of the objects frequently. However, children show more developed hand function with skillful executive function with aging. Although the exact reason for our finding remains to be clarified, from this known developmental process of motor function, we hypothesized that main location of CST might be changed progressively with aging, related to the development of function and maybe enlargement of PFC and PMC areas. Previous studies reported CST development is related to the development of clinical motor functions such as floundering to coordination and finally to executive function (Martin et al., 2004; Meng et al., 2004; Martin, 2005, 2006).

We demonstrated the significant anatomical change of CST with aging at cortex and CR level, and significant correlation of CST location from anterior to posterior direction with aging. To reveal more meaningful developmental process, the authors recruited subjects with wide ranges of age covering from 2-month-old infant to 19-year-old adolescent. All subjects were recruited according to the strict inclusion criteria and were evaluated and confirmed as normally, typically developing subjects by an expert neurologist for the accuracy of this study. In addition, all results showed high test–retest reliability. However, several limitations should be also considered. The major potential limitation of this study was the partial volume effect due to the same acquisition parameter for the DTI in all age. The partial volume effect can cause the underestimation of the fiber volume in younger child subject compared with adult subject. Second, regions of fiber complexity and crossing fibers can prevent full reflection of the underlying fiber architecture (Parker and Alexander, 2005; Yamada, 2009). Third, detailed and identical clinical data could not be obtained due to various distributions of age in subjects. Lastly, we did not separate SMA and PMC. Our study includes some very young subjects such as 2∼4 months’ old infants whose boundary between SMA and PMC was not obvious. It is another important limitation. The aim of this study was to demonstrate the maturational direction such as anterior to posterior, so the authors defined boundaries according to the direction from anterior to posterior such as PFC, PMC, M1 and S1. Due to these different boundaries of ROIs, direct comparison of these results with previous studies is impossible. The narrow projection of CST in our study with immature brain can be the different point compared to other studies with older subjects or adults. Therefore, further studies to overcome these limitations such as larger studies including varying age groups and more specific developmental process of CST on human brain are warranted.

## Author Contributions

SS and JR designed research; YK and HK performed research; SS and YK wrote the paper.

## Conflict of Interest Statement

The authors declare that the research was conducted in the absence of any commercial or financial relationships that could be construed as a potential conflict of interest.

## Footnotes

1 www.fmrib.ox.ac.uk/fsl
